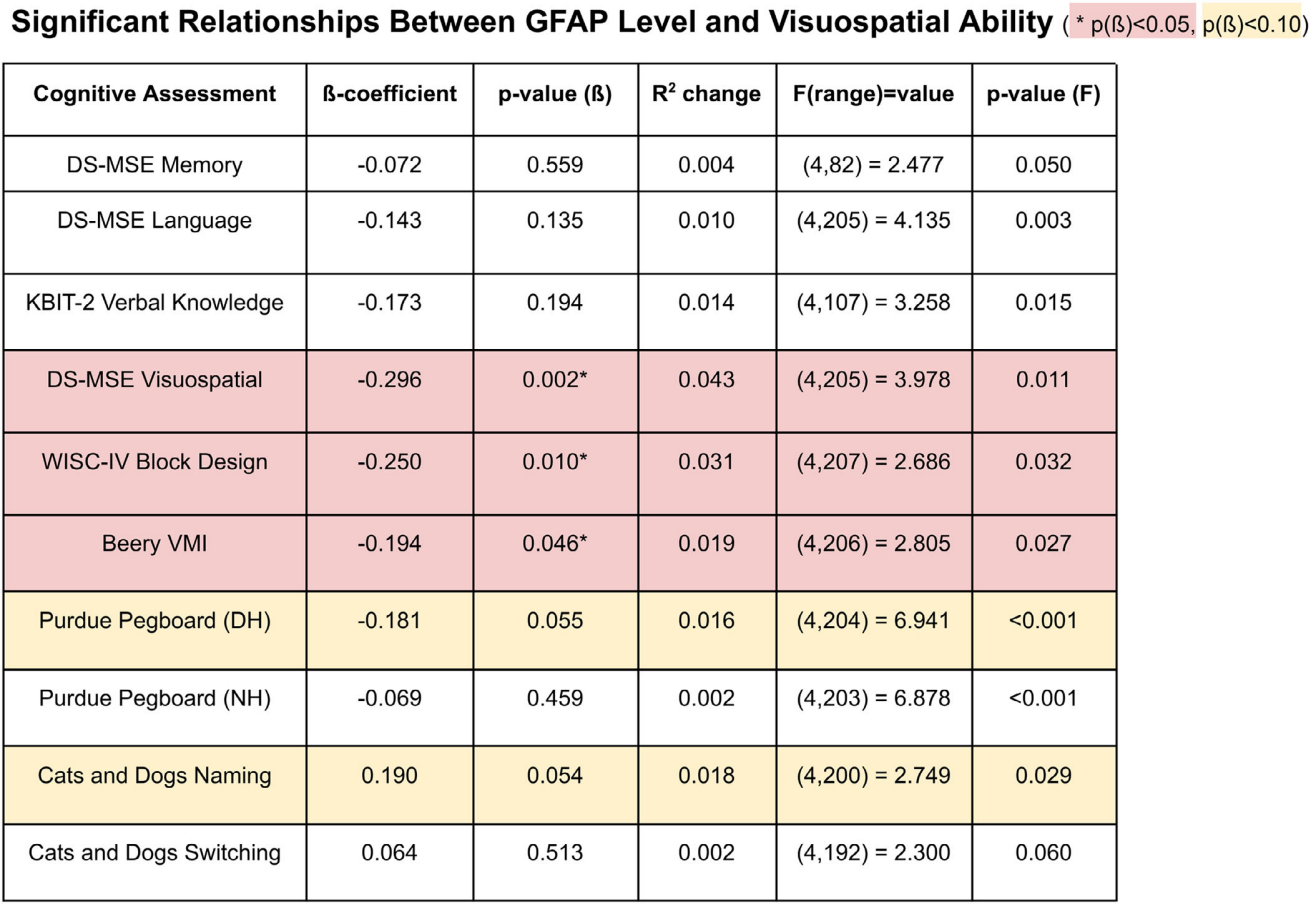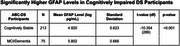# Glial fibrillary acidic protein as a biomarker for visuospatial ability in Down syndrome

**DOI:** 10.1002/alz70861_108368

**Published:** 2025-12-23

**Authors:** Anna Kiesewetter, Jennifer Bruno

**Affiliations:** ^1^ Stanford University, Stanford, CA USA; ^2^ Stanford, Palo Alto, CA USA

## Abstract

**Background:**

Down syndrome (DS) may serve as a model to understand biological factors modifying aging and cognitive decline, given the high incidence of Alzheimer's disease (AD) in individuals with DS. Glial fibrillary acidic protein (GFAP) is the plasma biomarker that displays the highest fold change in dementia participants compared to asymptomatic patients. Given that higher GFAP load is associated with amyloid PET uptake in both individuals with DS and AD, studying its correlations with cognitive ability in DS may pinpoint a cost‐effective, accessible biomarker to assist diagnosis and monitoring of AD‐related cognitive impairment.

**Methods:**

We examined the association between plasma GFAP levels and cognitive ability using data from the Alzheimer’s Biomarker Consortium in Down Syndrome (ABC‐DS). Participants included 231 cognitively stable adults with DS and 75 DS adults with mild cognitive impairment (MCI) or dementia. We ran a t‐test to compare mean GFAP levels in cognitively stable adults to MCI/dementia adults with DS cross‐sectionally. We then used hierarchical regression to test the relationship between GFAP and cognitive assessment scores, including memory via the Down Syndrome Mental Status Examination (DS‐MSE), language via the DS‐MSE and Kaufmann Brief Intelligence Test, visuospatial skills via the DS‐MSE, Wechsler Intelligence Scale for Children (WISC‐IV) Block Design, Beery Visual‐Motor Integration (VMI), and Purdue Pegboard, and executive function via the Cats and Dogs Task. We controlled for sex, APOE‐4 allele status, and age.

**Results:**

Mean GFAP levels were significantly higher in individuals with MCI/dementia compared to cognitively stable individuals. Higher levels of plasma GFAP were significantly associated with lower scores on cognitive assessments measuring visuospatial ability in cognitively stable individuals, including DS‐MSE Visuospatial Skills, WISC‐IV Block Design, and Beery VMI. Although higher levels of GFAP were correlated with lower scores on other assessments analyzed, these associations were no longer significant when accounting for age.

**Conclusion:**

These preliminary results indicate that GFAP may be a useful plasma biomarker for visuospatial ability level in adults with DS. Continuing this research holds potential for enhancing early detection of AD‐related cognitive impairment. Future analyses should assess correlations longitudinally and compare correlations to those of DS individuals with cognitive impairment.